# Urinary transforming growth factor beta-1 levels correlate with the effect of renorrhaphy on functional outcomes post-laparoscopic partial nephrectomy: A pilot-study

**DOI:** 10.1080/20905998.2024.2432702

**Published:** 2024-11-25

**Authors:** Aly M. Abdel-Karim, Ahmed M. Bakr, Mustafa A. Shamaa, Mokhtar A. Metawee, Ahmed I. El-Sakka

**Affiliations:** a Department of Urology, Alexandria University, Alexandria, Egypt; b Department of Urology, Suez Canal University, Ismailia, Egypt

**Keywords:** Functional outcome, healing, partial nephrectomy, renal cell carcinoma, fibrosis, tumor bed

## Abstract

**Purpose:**

Preservation of functional renal parenchyma is one of the main targets of partial nephrectomy. We investigated the effects of suture on renal parenchyma in tumor bed and on short-term renal function.

**Materials and Methods:**

Patients with unilateral cT1 renal masses candidate for laparoscopic partial nephrectomy (PN) have been recruited. After tumor excision, medullary sutures were replaced by argon beam in Group 1, while Group 2 had conventional 2-layer renorrhaphy. Groups have been matched using propensity score. Transforming growth factor beta-1 (TGFb1) levels in urine have been measured at the 1^st^ and 30^th^ day post-PN. Glomerular filtration rate has been estimated (eGFR) at baseline and 3 months post-PN.

**Results:**

Sixteen cases were matched in each group. There was no difference between groups regarding baseline, operative and perioperative data. Number of sutures in group 1 is nearly half that in group 2 (10 vs 19, respectively, *p* < 0.001). Group 1 showed lower urinary TGFb1 levels at the 1^st^ and 30^th^ day post-PN (*p* < 0.01 for each), higher eGFR after 3 months (*p* = 0.01), and less decline of eGFR from baseline (*p* = 0.046).

**Conclusion:**

TGFb1 levels in urine after PN are related to the number of sutures. Reduced number of sutures in tumor bed has a positive effect on short term eGFR changes possibly by reducing tumor bed fibrogenic healing response as well as preserving renal parenchymal volume.

## Plain Language Summary

After excision of renal tumors, surgeons should reduce the number of sutures in tumor bed to reduce the amount of scar and improve the post-operative renal function.

## Introduction

Partial nephrectomy (PN) is the standard of care for T1 renal mass. The aim of PN is to preserve as much normal renal tissue as possible to prevent post-operative acute renal failure, chronic kidney disease (CKD) and associated metabolic and cardiovascular complications [1]. Suture renorrhaphy is the standard technique for hemostasis in the tumor bed after tumor excision [2]. This technique consumes ischemia time, involves a non-ignorable part of the functional parenchyma [2,3], and requires advanced laparoscopic skills [1].

Renal function is a good indicator for vital renal units but could be affected by the contralateral function or by a systemic disease [4,5]. Transforming Growth Factor Beta 1 (TGFb1) has shown a role in the pathogenesis of different urologic diseases, particularly renal fibrosis [6,7]. TGFb1 level can be linked to renal function changes when comparing different hemostatic techniques [8].

Preserving renal parenchyma has shown more important role than ischemia time [9,10]. Use of surface coagulation can reduce the amount of devitalized renal parenchyma that would be otherwise got encroached between sutures and suffer from chronic ischemia and fibrosis [2,3]. Argon beam coagulation (ABC) has shown good hemostasis of tumor bed when substituting deep (medullary) layer of hemostatic sutures [11], and it reduces ischemia time (WIT) and tissue damage [12,13].

The previous data encouraged us to compare the levels of TGFb1 in urine when ABC was used in place of the medullary renorrhaphy layer versus using the conventional 2-layer-renorrhaphy. The ultimate goal of the current study is to quantify the amount of devitalized renal tissue due to hemostatic renorrhaphy and predicting post-PN renal function using a feasible biomarker test.

## Patients and methods

### Study design

This is a propensity score-matched comparison study. Patients with cT1 single non-hilar renal mass were included, either having 2 functioning kidneys, a solitary kidney, or having CKD. Cases with absolute contraindication for laparoscopic surgery (e.g. sever cardiopulmonary insufficiency, and chronic obstructive lung disease) were excluded. After laparoscopic partial nephrectomy, Group 1 had ABC in-place of medullary renorrhaphy layer plus cortical suture renorrhaphy, and Group 2 had conventional 2-layer-suture renorrhaphy.

### Surgical procedures

Patients were positioned in modified lateral kidney position. Dissection to the renal hilum was done and the tumor was then exposed. Clamping of renal artery only using laparoscopic Bulldogs. After excision of the mass, ABC was used to spray the tumor bed in Group 1. The cortical layer was sutured with Vicryl 2/0 and secured with Weck hem-o-lok clips (sliding clip technique). In Group 2, renorrhaphy was done using similar techniques in medullary and cortical layers. Early unclamping was done after securing the medullary layer.

### Study outcomes

The number of sutures is the count of times the needle comes-out from the renal parenchyma. Renal function was estimated by serum creatinine and eGFR. eGFR was estimated using Modification of Diet in Renal Disease equation [14]. Change in eGFR has been calculated in relation to baseline pre-operative levels, and a negative charge represents a decline in eGFR. TGFb1 level in urine (in ng/ml) were estimated using Sandwich-ELISA technique. Samples collected in the 1^st^ day and 30^th^ day postoperative have been investigated in this study.

### Statistical analysis

Analysis was conducted using IBM SPSS® statistics package version 22. Groups were matched using propensity score. A predicted probability was estimated for each case by logistic regression to Charlson index, tumor T stage, tumor size, and RENAL complexity. Matching was performed manually using nearest-neighbor technique. Student’s t-test was used to compare normally distributed continuous variables, otherwise, Mann-Whitney U test was used. To compare matched groups regarding post-operative eGFR and levels of TGFb1, we used Wilcoxon signed rank test. To compare the changes in repeated measures between groups, we used repeated measure ANOVA. Univariate regression analysis has been done. The Chi-squared test and Fisher’s exact were used to compare percentages. P-value was considered significant if <0.05. Ethical approval was taken from the local institutional research and ethical committee.

## Results

### Baseline data

Thirty-seven patients were recruited: 16 in Group 1 (ABC group) and 21 in Group 2 (Renorrhaphy group). Sixteen cases from each group were matched using their corresponding predicted probability. There were no significant differences between matched groups regarding baseline patient, functional and tumor features (*p* > 0.05 for each) (Table 1).

**Table 1.** Baseline characteristics of the studied groups.

Feature	Group 1	Group 2	p-value
	(n = 16)	(n = 16)	
**Patient features**			
**Age in years; mean ± SD**	50.1 ± 10.5	55.3 ± 6.5	0.1*
**Sex**			
Male; n(%)	8 (50)	7(43.8)	0.723**
Female; n(%)	8 (50)	9(56.3)	
**BMI; median (IQR)**	33.1(9.3)	34.3(9.5)	0.31^^^
**Comorbidities**			
Hypertension; n(%)	8 (50)	9(56.3)	0.723**
Diabetes; n(%)	3 (18.8)	2(12.5)	1^$^
**Charlson Comorbidity Index; mean ± SD**	3.1 ± 1	3.3 ± 0.6	0.4*
**Functional features**			
**Preoperative serum creatinine in mg/dl; median (IQR)**	1.2(0.2)	1.3(0.3)	0.34^^^
**Preoperative eGFR in mL/min; median (IQR)**	64.9(18.5)	58.4(18.1)	0.32^^^
**Tumor features**			
**Tumor size in mm: median (IQR)**	35.5(9.3)	35(7.3)	0.56^^^
**Side**			
Right; n (%)	5(31.3)	7(43.8)	0.465**
Left; n (%)	11(68.8)	9(56.3)	
**Tumor Stage**			
T1a; n (%)	15(93.8)	16(100)	1^$^
T1b; n (%)	1(6.3)	0	
**RENAL score; mean ± SD**	4.7 ± 0.95	4.8 ± 0.91	0.71*
**Pathology; n (%)**			
Malignant	12(75)	12(75)	1^$^
Benign	4(25)	4(25)	

IQR: interquartile range, *Student t-test; **Chi-squared test; ^Mann Whitney-U test; $ Fisher Exact.

### Operative outcomes

Operative and postoperative outcomes were comparable between groups (Table 2). Number of sutures and time of suturing in Group 1 were nearly half those in Group 2 (*p* < 0.001 for each). There was no conversion to open surgery, intraoperative or postoperative complications, no evidence of positive surgical margin, and no radiologic evidence of recurrence during follow-up duration (range 6–12 months).

**Table 2.** Comparison between groups in operative, postoperative and functional outcomes.

Features	Group 1 (*n* = 16)	Group 2 (*n* = 16)	p-value
**Operative and postoperative outcomes;** median (IQR)			
Operative time in minutes	128.5(34)	147(50.3)	0.184*
Ischemia time in minutes	13(10)	17(7.5)	0.11*
Estimated blood loss in ml	90(47.5)	100(15)	0.051*
Length of hospital stay in days	2 (1)	2 (1.8)	0.9*
**Tumor bed management;** median (IQR)			
Number of sutures	10 (4.3)	19 (12.5)	<0.001^*^
Time of renorrhaphy in minutes	14.5(7)	31.5(23.3)	<0.001^*^
**Urine TGFβ1 levels** (in ng/dl)			
At 1-day post-operative; median (IQR)	185(60)	300(44)	0.005**
At 30 days post-operative; median (IQR)	14(9)	24(46)	0.009**
**Functional outcomes** (eGFR in mL/min)			
eGFR at 3 months; median (IQR)	72(27.5)	56.5(5.5)	0.01**
Change in eGFR from baseline; mean±SD	−0.4 ± 20.5	−6 ± 5.9	**0.046**^**$**^

IQR: inter-quartile range; *Mann-Whitney U test; **Wilcoxon signed rank test; $ Repeated measures ANOVA.

### TGFb1 levels

**Figure 1.**
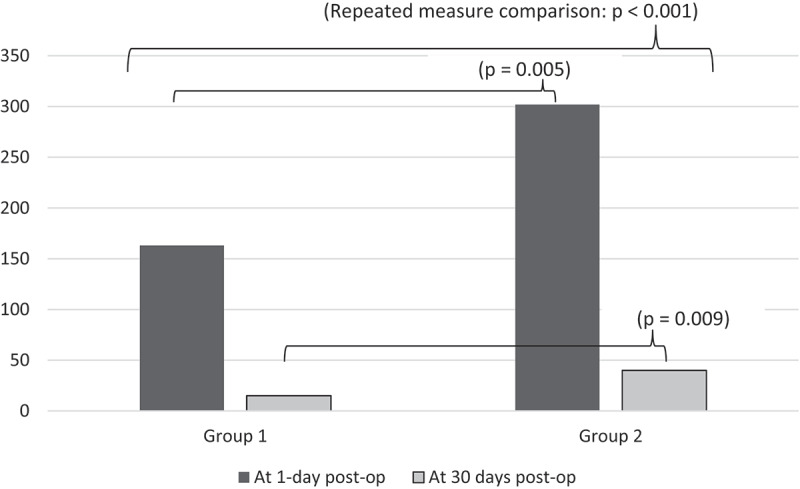
Comparing between study groups regarding levels of TGFb1 in urine at 1-day and 30 days after partial nephrectomy (*n* = 32).

Levels of TGFb1 showed a *tendency to decline* when comparing levels over time, in both groups (repeated measure comparison: *p* < 0.001) (Figure 1). Post-PN, levels were lower in Group 1 vs Group 2 at the 1^st^ day (*p* = 0.005), and the 30^th^ day (*p* = 0.009) (Table 2). Univariate regression analysis showed a significant relation of TGFb1 levels to number of sutures in the whole cohort (B = 2.2, *p* < 0.001).

### Functional outcomes

At 3 months post-PN, the median eGFR was significantly higher in Group 1 (*p* = 0.01). The mean eGFR declined in both groups after 3 months in comparison to baseline, but the decline of in Group 2 is significantly much more than that in Group 1 (*p* = 0.046) (Table 2).

## Discussion

PN has been extensively investigated in the last few decades [15]. The indications of PN extended from imperative to elective cases and have been recommended for the majority of T1 renal masses. The cumulative experience in tumor excision and the favorable oncologic and functional outcomes encouraged further extension of PN to include larger and more complex tumors [16]. One major concern with PN is the need to reduce postoperative morbidities by minimizing ischemia time and preserving normal renal parenchyma [17]. There is growing evidence that outweighs preservation of renal parenchyma over the reduction of ischemia time [9,18,19].

To our knowledge, this study is the first that discusses the possible effects of healing process in the tumor bed after PN. Intuitively, hemostasis and healing are two events actively ongoing in the tumor bed post-PN. The hemostatic effect of sutures depends mainly on their tension to overcome the blood pressure inside the vessels, which could concomitantly devitalize that segment [2,20]. Healing is the tissue response to injury, and several types of injurious events take place at the level of tumor bed in PN. Some injuries occur once intra-operatively, e.g., sharp dissection at the plane between tumor and parenchyma. Another, like renorrhaphy sutures, create a chronic ischemic injury that causes extended postoperative effects [21]. Deposition of extracellular matrix (ECM) or fibrosis is an important type of healing response in the kidney, which can impair the function of the affected renal parenchyma, and TGFb1 is a pivotal mediator in the process of renal fibrosis [8].

In our study, all operative outcomes were similar between groups, except the number of sutures in the tumor bed. Accordingly, the parenchyma of the kidney can be divided into 2 zones, the tumor bed, and the rest of the renal parenchyma. Warm ischemia affects most of renal parenchyma, while the tumor bed is additionally injured by the renorrhaphy sutures [13,22]. To minimize injury to this particular segment, we replaced the medullary layer with surface coagulation using ABC, in a way that balances between safety and efficacy [2,3].

In this study, TGFb1 levels measured in urine at the 1^st^ and the 30^th^ day postoperative have been investigated. Preoperative TGFb1 levels showed no difference between groups and there were no significant correlations with any baseline factors, particularly comorbidities and pathologic diagnosis of the tumor, similar to what has been reported by Sauriasari and his colleagues [23].

At the 30^th^ day post-PN, the average levels of TGFb1 declined dramatically in both groups, compared to levels at 1^st^ day. The levels in Group 1 were lower than Group 2. A strong positive relation between TGFb1 levels and number of sutures was found by univariate linear regression. That supports our assumption that sutures could induce a fibrogenic response at the tumor bed [2]. Patients in Group 2 with higher TGFb1 levels at the 30^th^ day showed lower eGFR after 3 months and suffered more decline from baseline eGFR.

We think that TGFb1 levels at 30^th^ day are determined by the rate of its release and by the time factor, i.e., the duration at which the ‘healing process’ settles and the active release of inflammatory mediators ceases. It seems that it takes longer to decline in patients who have more sutures in tumor bed. As it gets reflected on ultimate functional outcome, patients with persistently high TGFb1 levels at the 30^th^ day post-PN is anticipated to have worse eGFR at 3 months post-PN.

We succeeded in achieving hemostasis of the tumor bed in Group 1 by replacing medullary sutures with ABC. The median time of ABC was 2 min. The number of sutures has been reduced by nearly 50% in Group 1 in comparison to Group 2. On the other hand, the amount of estimated blood loss and the rate of blood transfusion (0%) were similar to conventional technique. There were no postoperative hemorrhage or urine leakage at either group, indicating safety but also omitting any possible sources for a fibrogenic process out of tumor bed.

Our argument is based on two pieces of evidence. First, that eGFR at 3 months post-PN can predict renal function status through the first 10 years after PN [24], and serum creatinine level could not be a reliable index for monitoring renal function post-PN [25]. Second, a systemic review of more than 80 trials that emphasized the favorable influence of reducing number of renorrhaphy sutures on the functional outcomes and functional recovery [2]. As shown in Group 1, those favorable functional outcomes were associated with a significant difference in urinary TGFb1 levels.

The effect of healing or fibrogenic process in the tumor bed after PN has not been clearly addressed. The attenuation of renal tissue response to the surgical injury by the reduction in number of renorrhaphy sutures has been referred to by Bertolo et al [2]. Our study supports the role of preserving functional renal tissue as being more important than ischemia time, in parallel to several studies [9,10,26]. We also provided evidence that extension of renorrhaphy has a non-favorable effect on functional outcomes post-PN and that reducing number of sutures has positive influence on eGFR, which was also recommended by many authors [2,21].

Our study provided TGFb1 as a marker for healing process in the tumor bed after PN and paved the way for further investigations of the therapeutic uses of TGFb1 post-PN. Possible implications include targeting TGFb1 signaling pathways to reduce the fibrogenic process and improve the final eGFR. On the other hand, we introduced the phenomenon of healing of tumor bed post-PN as a considerable factor to preserve renal function post-PN, side by side with renal parenchyma volume and ischemia time. Our main limitation was small sample size, which was due to restricted patient recruitment by a specific range of tumor size and complexity. We used eGFR to measure functional outcomes, and while it is widely accepted for that purpose [2], renographic split renal function could more precisely emphasize the effects of renorrhaphy [27].

In summary, fibrosis of tumor bed could contribute to the final functional outcomes after PN. One important measure to reduce fibrosis in tumor bed is by reducing the number and extension of sutures during renorrhaphy.

## Conclusion

TGFb1 levels in urine after PN are related to the amount of devitalized renal parenchyma due to renorrhaphy of tumor bed and can predict unfavorable renal function post-PN. Reducing the number of sutures in tumor bed has a positive effect on short term eGFR changes possibly by reducing tumor bed fibrogenic healing response as well as preserving renal parenchymal volume.
